# Integrated Physiological and Transcriptomic Analyses Suggest Key Adaptive Mechanisms of European Perch (*Perca fluviatilis*) to Acute Heat Stress

**DOI:** 10.3390/ani16132007

**Published:** 2026-07-01

**Authors:** Geng Chen, Fangyuan Peng, Peng Chen, Jin Xu

**Affiliations:** 1 Yangtze River Fisheries Research Institute, Chinese Academy of Fishery Sciences, Wuhan 430223, China; chengeng@yfi.ac.cn (G.C.);; 2 Freshwater Fisheries Research Center, Chinese Academy of Fishery Sciences, Wuxi 214081, China; 3 Xinjiang Uygur Autonomous Region Institute of Fishery Sciences, Urumqi 830099, China

**Keywords:** *Perca fluviatilis*, heat stress, endoplasmic reticulum stress, energy triage, apoptosis, transcriptome

## Abstract

The European perch (*Perca fluviatilis*) is highly susceptible to heat stress, limiting its sustainable aquaculture. While single-organ thermal responses are partially understood, the systemic, multi-organ cooperative survival mechanisms under acute heat stress remain poorly characterized. To elucidate the underlying tolerance mechanisms and provide genetic markers for breeding, this study investigated the multi-organ responses of European perch (*n* = 90; body length: 13.15 ± 1.75 cm; body weight: 30.54 ± 7.17 g) transferred from 24 °C to an acute heat stress challenge (31 °C) at an increasing rate of 2 °C/h, and the histopathological changes (liver and gill), hepatic biochemical biomarkers (CAT, SOD, GSH-Px, GST, LDH, and MDA), and transcriptomic changes (liver and kidney) were evaluated over a 24 h period. Heat stress induced progressive structural damage, including gill lamellar edema and hepatocyte necrosis, accompanied by significant hepatic oxidative stress and lipid peroxidation. RNA-seq transcriptome profiling uncovered distinct sets of genes with significant expression changes, comprising 1343 DEGs in liver tissue and 722 DEGs in kidney samples. Both organs shared a systemic endoplasmic reticulum stress response but exhibited highly divergent survival strategies. The liver underwent severe metabolic reprogramming towards anaerobic glycolysis and gluconeogenesis, coupled with vesicle-mediated membrane repair attempts and apoptosis. Conversely, the kidney adopted a strict “energy triage” strategy, suppressing highly energy-consuming immune and osmoregulatory functions while actively silencing pro-apoptotic signals. These findings highlight organ-specific adaptations and identify potential metabolic markers for the future breeding of new heat-tolerant varieties.

## Simple Summary

Global climate change and the increasing frequency of summer heatwaves pose a severe threat to the farming of the European perch, a highly valued cool-water fish. When water temperatures rise excessively, this species experiences life-threatening thermal stress. This study aimed to characterize how the vital internal organs of the perch respond to sudden, extreme heat. Our findings indicate that high temperatures induce severe respiratory distress and impaired oxygen uptake due to structural gill damage. To cope, the perch dramatically remodels its energy metabolism. The liver attempts to initiate massive cellular repair but ultimately sustains severe pathological damage. Concurrently, the kidney demonstrates evidence of an energy-conservation strategy by temporarily suspending non-essential physiological functions to conserve basal cellular resources. While both organs experience profound protein disruption, they display divergent cellular responses: damaged hepatocytes tend to undergo cell death, whereas renal cells strongly resist apoptotic signaling to prevent total organ failure. Ultimately, although the perch struggles against the heat, prolonged exposure overwhelms its physiological defenses.

## 1. Introduction

Worldwide, climate change is escalating both the frequency and severity of summer heatwaves, which pose grave risks to freshwater aquaculture industries [1]. Water temperature acts as a master environmental variable that strictly dictates the physiological limits, metabolic performance, and survival of ectothermic fishes [2]. Given that modern intensive aquaculture relies on stable aquatic conditions, abrupt thermal extremes both limit the geographic range of suitable culture areas and impair fish welfare and production. This thermal stress has turned into a major barrier restricting sustainable industrial expansion [3]. The European perch (*Perca fluviatilis*) is a cool-water teleost naturally distributed throughout Europe and northern Asia with an optimal growth temperature range of 22–24 °C. It has important ecological functions and high economic value, making it a prime candidate for intensive aquaculture [4,5]. Within China, it is indigenous exclusively to the Irtysh and Ulungur River basins of Altay Prefecture, Xinjiang Uygur Autonomous Region [6]. The global total production of *P. fluviatilis* reached 34,800 tonnes in 2024 according to FAO FishStat fishery statistics [7]. Specifically, while the global farmed output of European perch officially exceeded 500 tonnes for the first time in 2013, the commercial artificial breeding of this species remains at an early industrial stage. The up-scaling of intensive European percid fish culture continues to face critical technical bottlenecks. As extensively documented in recent global aquaculture assessments, modern percid farming is severely constrained by high early-stage mortality, metabolic stress under high-density rearing, and an extreme vulnerability to fluctuating environmental parameters [8,9]. In China, the natural distribution and farming of *P. fluviatilis* are primarily concentrated in the specific basins of the Xinjiang Uygur Autonomous Region, and the domestic breeding scale is also limited compared with mainstream cultured freshwater species [10]. Unlike the cool climate in Xinjiang, surface water temperatures of culture ponds in central and southern China regularly exceed 32 °C during summer [11,12]; as *P. fluviatilis* is a cold-water fish optimally reared at 22–24 °C, these high temperatures severely constrain the expansion of its aquaculture to other regions of China.

Fish in warm water suffer from severe physiological dysfunctions, including compromised respiration, imbalanced metabolism, and depleted energy reserves [13]. Because gills are continuously exposed to water and serve as the main gas-exchange organ, they readily develop structural impairments under thermal stress [14]. Elevated temperatures cause lamellar edema and epithelial lifting, which significantly thicken the diffusion barrier, impair oxygen uptake, and may potentially induce systemic hypoxia [15]. Second, this hypoxia-thermal combined crisis forces a massive metabolic reprogramming in central metabolic organs like the liver. To cope with the surging energy demands of cellular repair while oxygen is limited, the liver is forced to shift from aerobic respiration to anaerobic glycolysis and gluconeogenesis, rapidly disrupting metabolic homeostasis [16]. Finally, confronted with this severe energetic bottleneck, fish must strategically reallocate their limited physiological resources—prioritizing essential somatic maintenance over growth and reproduction through a process of metabolic triage [17,18]. As the key organ responsible for osmoregulation and innate immunity, the kidney likely undergoes adaptive functional suppression to conserve energy under extreme heat stress [19].

At the cellular level, oxidative stress and endoplasmic reticulum (ER) stress are universal damage mechanisms employed by organisms subjected to extreme temperatures [20]. Thermal stimulation speeds up organismal metabolic activity and disturbs the operation of mitochondrial electron transport chains, disrupting the dynamic equilibrium of reactive oxygen species (ROS) generation and clearance inside cells [13]. Excess ROS attack biomacromolecules, causing severe lipid peroxidation, indicated by elevated malondialdehyde (MDA) levels, and leading to cellular functional impairment [21]. Although organisms mobilize antioxidant defense systems—including Superoxide Dismutase (SOD) and Catalase (CAT)—to scavenge ROS, this defense is often overwhelmed by prolonged lethal heat exposure [13]. Concurrently, accumulated heat-denatured proteins induce severe endoplasmic reticulum (ER) stress and activates the Unfolded Protein Response (UPR) [22]. Although the UPR is initially activated to reestablish cellular protein homeostasis, persistent and unresolved ER stress rewires cell fate toward apoptosis, thereby causing irreversible tissue injury [20].

Conventional phenotype-focused research cannot clarify the molecular basis of thermal stress adaptation; whole-transcriptome RNA sequencing thus serves as an effective analytical strategy to address this limitation. Through capturing genome-wide transcriptional profiles, RNA-Seq decodes sophisticated interactive molecular pathways linked to heat stress damage and compensatory adaptive reactions [23]. Currently, transcriptomic approaches have been widely applied to investigate heat stress responses in various commercially important teleosts, such as rainbow trout (*Oncorhynchus mykiss*), Chinese tongue sole (*Cynoglossus semilaevis*), and grass carp (*Ctenopharyngodon idella*) [24,25,26].

Despite the immense ecological significance and escalating aquaculture value of the European perch, our comprehensive understanding of its specific physiological and molecular limits under acute thermal stress remains incomplete and fragmented. Historically, large-scale field monitoring has consistently shown the species’ high sensitivity to climate-driven temperature fluctuations, where extreme thermal variation is a primary driver of year-class strength, recruitment success, and long-term population dynamics in natural waters such as the Baltic Sea [27]. At the physiological level, recent laboratory assessments have revealed that while chronically acclimated perch exhibit limited cardiorespiratory plasticity to cope with elevated baseline temperatures, their upper critical thermal maximum (CTmax) and cardiac stroke volume limits remain relatively inflexible, which effectively constrains their available “thermal buffer” capacity when facing sudden environmental temperature spikes [28,29].

However, the molecular mechanisms underlying these observed physiological responses remain largely uncharacterized in *P. fluviatilis*. For comparison, in thermally tolerant cyprinids such as grass carp (*C. idella*), studies have documented robust and sustained systemic antioxidant compensatory responses to cope with heat stress [30]; in cold-water salmonids such as rainbow trout (*O. mykiss*), acute heat exposure typically triggers strong immune-inflammatory cascades [31]. In contrast, the specific molecular regulatory networks and multi-organ energetic trade-offs that determine the acute thermal survival limits of *P. fluviatilis* have yet to be systematically elucidated across multiple vital tissues.

To address these research gaps, the present study aimed to: (1) characterize the temporal dynamic changes in histopathological lesions in heat-sensitive gill and liver tissues; (2) employ biochemical testing to profile dynamic hepatic biochemical fluctuations, with special emphasis on oxidative stress status; (3) leverage dual-organ transcriptomics to dissect molecular regulatory responses underlying heat stress adaptation in the liver and kidney; and (4) integrate histological, biochemical, and transcriptomic data to construct a comprehensive pathophysiological model of acute heat stress. This study aims to deepen the understanding of organ-specific thermal adaptation mechanisms and provide reliable molecular markers for the selective breeding of heat-tolerant perch strains.

## 2. Materials and Methods

### 2.1. Experimental Animals and Acclimation

All animal experiments were approved by the Ethics Committee of the Yangtze River Fisheries Research Institute (protocol code: YFI2024CHG01). A total of 600 one-year-old healthy European perch were sourced from the Xinjiang Aquatic Wildlife Rescue Center for this study, with an initial body length range of 10–16 cm and body weight range of 20–40 g. Prior to all experiments, the fish were acclimated in 250 L circular fiberglass tanks with 1500W variable-frequency PTC heaters (Chuangning, Zhongshan, China) for two weeks at a constant optimal temperature of 20 ± 0.5 °C under a 12 L:12 D photoperiod. Water was recirculated through a biological filtration system with a 20% daily replacement rate. Dissolved oxygen was held higher than 6.0 mg/L during acclimation, and ammonia as well as nitrite were restricted to concentrations below 0.01 mg/L at all times. Fish were fed to visual satiation twice daily (2% of their body weight) with commercial pellets (Tongwei, Chengdu, China; 48% crude protein, 6% crude lipid), but were subjected to a 24 h fasting period immediately prior to the thermal stress trials to eliminate the confounding effects of digestion.

### 2.2. Thermal Tolerance Determination and Acute Heat Stress Modeling

To evaluate the thermal tolerance limits of *P. fluviatilis* and identify the optimal temperature for subsequent molecular analyses, we conducted a preliminary trial that included both gradual warming and acute exposure.

For the gradual warming model, designed to mimic the onset of natural summer heatwaves, healthy fish were randomly distributed into triplicate tanks (10 fish per tank, *n* = 30). The water temperature was initially adjusted from the 20 °C optimum to a typical early-summer baseline of 24 °C. Following a 72 h stabilization period at this baseline, the temperature increased from 24 °C to 33 °C at a rate of 1 °C/24 h. Daily survival was monitored to identify the onset of thermal lethality.

Differently, the acute thermal stress model revised to be consistent with the pre-experimental acclimation regime of the gradual warming group aimed to define the absolute physiological boundaries of fish under abrupt high-temperature exposure. All experimental fish were first acclimated at the early-summer baseline temperature of 24 °C for 72 h to adapt to the stable experimental water environment. Subsequently, fish were directly transferred to pre-configured target temperature water bodies ranging from 27 °C to 33 °C for acute stress treatment. For each target temperature group, fish were allocated into triplicate tanks (15 fish per tank, *n* = 45 per temperature group). No gradient heating process from 20 °C was adopted in this acute trial. After fish were transferred to the corresponding target temperature environments, cumulative mortality was recorded at 6, 12, and 24 h post-exposure. The integration of mortality profiles from both preliminary models clearly established 31 °C as the critical sublethal physiological precipice. At this specific temperature, the fish exhibited profound sublethal stress characteristics, yielding interval mortality rates of 13%, 7%, and 24% at 6, 12, and 24 h, respectively, culminating in a cumulative 24 h mortality of 44%. Approaching this near-median lethal threshold (LD50) ensures that the surviving population is operating at their maximal adaptive limit, providing optimal tissue samples for capturing definitive, life-or-death transcriptomic reprogramming without resulting in the total population collapse observed instantaneously at 32 °C and 33 °C.

Following the definitive establishment of this threshold, the formal acute heat stress experiment was initiated with a unified 24 °C pre-acclimation procedure consistent with the preliminary trials. A total of 90 thoroughly acclimated fish (body length: 13.15 ± 1.75 cm; body weight: 30.54 ± 7.17 g) were randomly allocated into three replicate stress tanks (30 fish per tank). All fish were first maintained at the 24 °C early-summer baseline temperature for 72 h of stable acclimation. The water temperature in the stress groups was gradually elevated from 24 °C to the target temperature of 31 °C at a steady rate of 2 °C/h [28,29], and subsequently maintained at 31 ± 0.5 °C for 24 h. A parallel control group of 30 fish was constantly maintained at 24 °C. Continuous aeration was provided to all tanks throughout the experimental period.

### 2.3. Tissue Sampling and Preservation

Tissue sampling was performed at 0 (control), 6, 12, and 24 h post-stress. At each designated time point, three fish per tank (3 tanks × 3 fish = 9 fish per time point) were randomly captured and immersed in tricaine methanesulfonate (MS-222, 0.03 g/L) for 3–5 min to achieve deep anesthesia. For the baseline control (0 h), samples were collected from a parallel, non-stressed cohort maintained continuously at 24 °C immediately prior to the onset of the acute thermal exposure protocols. Loss of opercular ventilation and absence of tail reflex were verified to ensure sufficient anesthetic depth prior to dissection to eliminate nociception. The liver, kidney, and gill tissues were rapidly excised on ice within 2 min to limit suffering. For histopathological evaluation, tissue fragments were immediately fixed in 4% paraformaldehyde. For biochemical enzyme assays, the liver aliquots were snap-frozen in liquid nitrogen and stored at −80 °C. For RNA extraction and transcriptomic analysis, the liver and kidney samples were initially stabilized in RNAlater (Thermo Fisher Scientific, Waltham, MA, USA) preservation solution at 4 °C for 24 h, followed by long-term storage at −80 °C. Following tissue harvest, all experimental individuals were humanely euthanized via immersion in an overdose of MS-222 solution. All fish carcasses were collected separately and delivered to the laboratory’s designated hazardous waste agency for standardized incineration treatment, consistent with approved animal welfare protocols.

### 2.4. Histopathological Processing and Evaluation

Liver and gill samples fixed in paraformaldehyde were subjected to routine histological processing. After complete fixation, tissues were dehydrated through a graded ethanol series, cleared with xylene, and embedded in paraffin wax. Tissue sections of 5 μm thickness were prepared using a rotary microtome, followed by standard hematoxylin and eosin (H & E) staining. Histopathological structures and lesions were observed and imaged with a Nikon ECLIPSE Ts2 microscope (Shinagawa, Japan).

### 2.5. Biochemical Assays for Hepatic Oxidative Stress and Metabolism

Hepatic metabolic reprogramming and cellular oxidative stress profiles were biochemically interrogated using properly prepared tissue homogenates. Specifically, cryopreserved liver fragments were mechanically dispersed within a chilled physiological saline medium maintained strictly at a 1:9 (*w*/*v*) ratio. To isolate the functionally active supernatant fractions, these crude homogenates were subjected to high-speed cold centrifugation (4 °C, 4000× *g*) for exactly 10 min. Following supernatant collection, absolute soluble protein concentrations were determined to enable accurate enzymatic normalizations, employing a standard Bicinchoninic Acid (BCA) protocol [32]. The quantitative mapping of the cellular defense array involved tracking the kinetic activities of primary antioxidant scavengers (SOD, CAT, and GSH-Px) alongside pivotal phase II detoxification and anaerobic metabolic operators (GST and LDH). Concurrently, the accumulation burden of MDA was evaluated to index the severity of lipid peroxidation. The entirety of these biochemical assessments was conducted utilizing proprietary colorimetric assay architectures supplied by the Nanjing Jiancheng Bioengineering Institute (Nanjing, China), with all analytical steps rigidly adhering to their optimized operational guidelines. Ultimate optical density recordings for the diverse panels were consistently read through a MAPADA UV1100 spectrophotometric instrument (Shanghai, China).

### 2.6. Isolation of Total RNA Extraction and High-Throughput Transcriptome Sequencing

TRIzol reagent was adopted to isolate total RNA from hepatic and renal specimens, with three independent biological replicates prepared for each tissue at both the 0 h non-stressed control and 24 h thermal challenge time points [33]. Two detection instruments, a NanoDrop spectrophotometer (Thermo Fisher Scientific, Waltham, MA, USA) and Agilent 2100 Bioanalyzer (Agilent Technologies, Santa Clara, CA, USA), were jointly adopted to thoroughly evaluate three key RNA indicators: purity, concentration and integrity. Specimens satisfying strict RNA quality thresholds (OD260/280 ranging from 1.9 to 2.1, RNA concentration exceeding 300 ng/µL) were retained to synthesize complementary DNA libraries. The finished cDNA libraries were then subjected to high-throughput sequencing on the Illumina NovaSeq 6000 sequencing system (Illumina, San Diego, CA, USA).

### 2.7. Bioinformatic Pipeline and Differential Expression Analysis

To guarantee the analytical fidelity of downstream gene expression modeling, the massive volume of raw sequencing outputs was initially funneled through a strict quality filtration pipeline powered by the fastp toolkit (v0.20.0) [34]. This preliminary sweep successfully excised ambiguous technical adapter contaminations and structurally substandard sequence reads, thereby securing a robust foundation of high-quality clean data. These refined read datasets were subsequently anchored directly against the highly contiguous structural assembly of the primary *P. fluviatilis* reference genome, capitalizing on the ultra-fast indexing and mapping algorithms inherent to HISAT2 [35,36]. For the objective standardization of global transcript accumulation, all genic expression profiles were mathematically normalized applying the widely accepted FPKM (Fragments Per Kilobase of transcript per Million mapped reads) quantitative metric [37,38]. The critical statistical extraction of differentially expressed genes (DEGs) induced by the extreme acute thermal loading was executed by deploying the negative binomial distribution models functioning within the DESeq2 analytical ecosystem [39]. A highly stringent dual-parameter filtering threshold was enforced to isolate biologically meaningful transcriptomic shifts: specific genes were statistically categorized as significantly regulated only upon demonstrating an adjusted False Discovery Rate (FDR) below 0.05, concurrently coupled with an absolute log2 fold change (FC) deviation strictly exceeding 1. To further decode the broader physiological and metabolic ramifications of these identified transcriptomic perturbations, the complete library of significant DEGs was systematically funneled into expansive functional annotation networks, leveraging both Gene Ontology (GO) categorizations and comprehensive Kyoto Encyclopedia of Genes and Genomes (KEGG) pathway enrichment algorithms [40,41].

### 2.8. Quantitative Real-Time PCR (qRT-PCR) Validation

The analytical fidelity and quantitative integrity of the high-throughput transcriptomic sequencing platform were rigorously corroborated via an independent series of quantitative real-time PCR (qRT-PCR) evaluations. Specifically, a targeted panel of eight distinct hallmark DEGs was meticulously tracked for sustained expression tracking across the entire 24 h progressive thermal challenge continuum. The foundational reverse transcription procedures, responsible for converting the pool of extracted total RNA into viable first-strand cDNA, were systematically driven by the robust PrimeScript™ RT reagent framework (TaKaRa, Shiga, Japan). Subsequent fluorescent amplifications targeting the selected genetic loci were fluorometrically quantified upon a dedicated real-time thermal cycling unit integrated with TB Green^®^ Premix Ex Taq™ (TaKaRa) chemistry. Crucially, to entirely eliminate potential normalization biases inherently associated with extreme elevated temperature stress conditions, an initial comprehensive stability screening protocol was performed across an array of four commonly utilized housekeeping candidates (specifically β-actin, EF1α, GAPDH and 18S rRNA). Because the β-actin transcript consistently demonstrated optimal baseline stability alongside negligible transcriptional fluctuation regardless of the applied thermal severity, it was officially validated and designated as the permanent internal baseline anchor for all relative expression adjustments. Final relative transcript dynamic outputs were computationally derived applying the universally standardized 2^−ΔΔCt^ comparative algorithmic equation [42]. Three technical replicates were performed for each biological sample during qRT-PCR. The complete suite of custom oligonucleotide amplification primers underpinning these assays (exhaustively detailed in Supplementary Table S1) was commercially designed and procured via Tianyi Huayu Co., Ltd. (Wuhan, China).

### 2.9. Statistical Analysis

The IBM SPSS Statistics (version 27.0; IBM Corp., Armonk, NY, USA) software ecosystem served as the central computational nexus for all quantitative assessments detailed throughout this manuscript. All quantitative data are expressed as the mean ± standard error (SE). To rigorously identify and delineate the significant temporal variations inherently induced by the progressive thermal exposure protocol, the cumulative dataset was subjected to a rigid analytical framework grounded fundamentally upon a one-way analysis of variance (ANOVA). In instances where significant primary overarching variance was statistically confirmed, Tukey’s specialized post hoc protocols were immediately deployed to execute highly granular multi-group inter-comparisons. Across the entirety of these inferential statistical models, the definitive threshold for confirming empirical statistical significance was rigidly demarcated at a critical alpha level of *p* < 0.05.

## 3. Results

### 3.1. Thermal Tolerance Thresholds and Mortality Dynamics Under Heat Stress

The survival rate of *P. fluviatilis* was influenced by both temperature and exposure time, exhibiting a clear threshold response.

**Figure 1:**
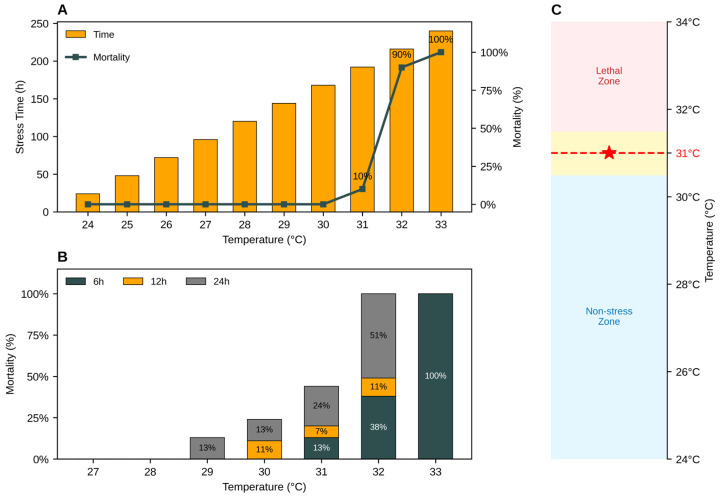
Rationale for experimental temperature selection in European perch (*Perca fluviatilis*) under thermal stress. (**A**) Cumulative mortality rate (%) and total stress duration (h) during the gradual warming experiment (24 °C to 33 °C, 1 °C/24 h). Data labels on the line indicate the cumulative mortality at critical temperature nodes. (**B**) Distribution of mortality rates in the acute thermal stress experiment across temperatures ranging from 27 °C to 33 °C. Stacked bars represent the cumulative mortality at 6 h (dark green), 12 h (orange), and 24 h (gray). Percentage values within each segment indicate the interval-specific mortality rate. (**C**) Schematic diagram of thermal tolerance zones. The blue, yellow, and red regions represent the non-stress zone (≤30.5 °C), sublethal stress zone (30.5–31.5 °C), and lethal zone (≥31.5 °C), respectively. The red dashed line and star mark the selected target stress temperature (31 °C).

In the acute thermal exposure, mortality increased in a stepwise manner with rising temperature and extended exposure time. Groups maintained at 27 °C and 28 °C exhibited high thermal tolerance with no mortality within 24 h. Sporadic mortality first occurred at 29 °C, with a 24 h cumulative mortality of 13%. At 31 °C, fish showed clear sublethal stress responses, clinically characterized by a progressive loss of equilibrium, severe lethargy, and vigorous surface gasping indicating severe respiratory distress, with interval mortality rates of 13%, 7%, and 24% at 6 h, 12 h, and 24 h, respectively, resulting in a cumulative mortality of 44% at 24h. Conversely, temperatures of 32 °C and 33 °C reached highly lethal, with 100% mortality occurring within 24 h; notably, the 33 °C group reached 100% mortality after only 6 h of exposure (Figure 1B). These results demonstrate the coupled effect of temperature and time on survival: mortality increased significantly with temperature at the same exposure duration, and rose progressively with longer exposure at the same temperature.

In the gradual warming assay, fish exhibited moderate thermal buffering capacity. The survival rate remained at 100% until the water temperature reached 30 °C (within the first 168 h of warming). The first occurrence of mortality (10% cumulative) was recorded at 31 °C, indicating the entry into a critical physiological stress state. Subsequently, cumulative mortality increased sharply, reaching 90% at 32 °C and 100% at 33 °C (Figure 1A). Compared with acute thermal exposure, gradual warming allowed for stepwise physiological acclimation, resulting in a higher thermal tolerance threshold, which reflects the regulatory effect of warming rate on thermal resistance.

By integrating mortality data from both acute and gradual warming assays, three thermal tolerance zones with distinct physiological characteristics were defined for *P. fluviatilis* (Figure 1C): (1) Non-stress tolerance zone (≤30.5 °C): Within this temperature range, *P. fluviatilis* maintains high survival rates over prolonged exposure. Homeostasis can be sustained via routine physiological regulation, without significant oxidative damage or histopathological alterations, representing the normal thermal tolerance range; (2) Sublethal stress zone (30.5–31.5 °C): This is the critical transition range of thermal tolerance. Mortality increases gradually with extended exposure time, and the compensatory capacity of antioxidant and metabolic systems approaches its limit. Significant physiological impairment and histopathological changes can be detected, but rapid mass mortality does not occur. This zone is suitable for investigating the mechanisms of thermal stress response; (3) Lethal collapse zone (≥31.5 °C): Temperatures in this range induce extremely high mortality within a short period. The physiological defense system declines rapidly, and severe irreversible damage occurs in cells and tissues, making it impossible to maintain basic vital activities.

Based on this zoning, 31 °C was selected as the target temperature for the formal thermal stress experiment. Located in the core of the sublethal stress zone, this temperature can induce robust physiological stress responses while ensuring sufficient surviving individuals for subsequent molecular and physiological assays, allowing for characterization of the critical regulatory mechanisms of *P. fluviatilis* under high temperature stress.

### 3.2. Time-Dependent Histopathological Lesions in the Liver and Gills

**Figure 2:**
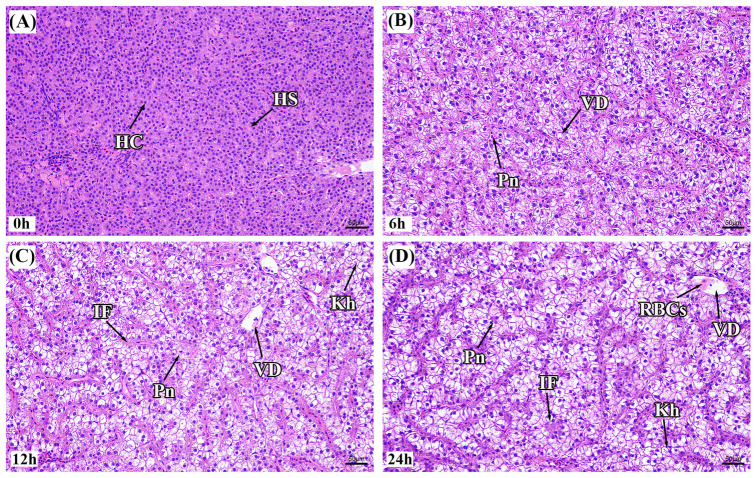
Hepatic histomorphological lesions induced by thermal stress in European perch (*Perca fluviatilis*) after H & E staining. (**A**) Liver sections from the 0 h control group display intact hepatocytes (HC) and regular hepatic sinusoids (HS). (**B**–**D**) Gradually aggravated tissue injuries observed at 6 h, 12 h and 24 h heat exposure, featuring cytoplasmic vacuolar degeneration (VD), nuclear pyknosis (Pn, hyperchromatic shrunken nuclei), karyorrhexis (Kh, disrupted nuclear structure), inflammatory cellular infiltration (IF), and intrahepatic erythrocyte stasis (RBCs). Scale bar = 50 μm.

HE-stained liver sections revealed an intact hepatic architecture in the control group of *P. fluviatilis*. Hepatocytes exhibited a regular polygonal morphology with uniformly dense cytoplasm and no evident vacuolation. The cells were arranged in an orderly pattern, featuring centrally located nuclei and compact hepatic sinusoids (Figure 2A). With prolonged exposure to elevated temperature, the liver tissue displayed progressively severe morphological alterations. After 6 h of heat stress, hepatocyte nuclei showed signs of pyknosis, cell boundaries appeared indistinct, and mild congestion along with focal vacuolation was observed. Slight dilation of the hepatic sinusoids and perisinusoidal spaces was also evident (Figure 2B). Following 12 h of exposure, marked nuclear displacement and pronounced inflammatory cell infiltration were apparent, accompanied by significant expansion of the sinusoids (Figure 2C). After 24 h of continuous high temperature treatment, hepatocyte nuclei were fragmented or dissolved, the native cellular architecture was disrupted, and the hepatic sinusoids were extensively dilated (Figure 2D).

**Figure 3:**
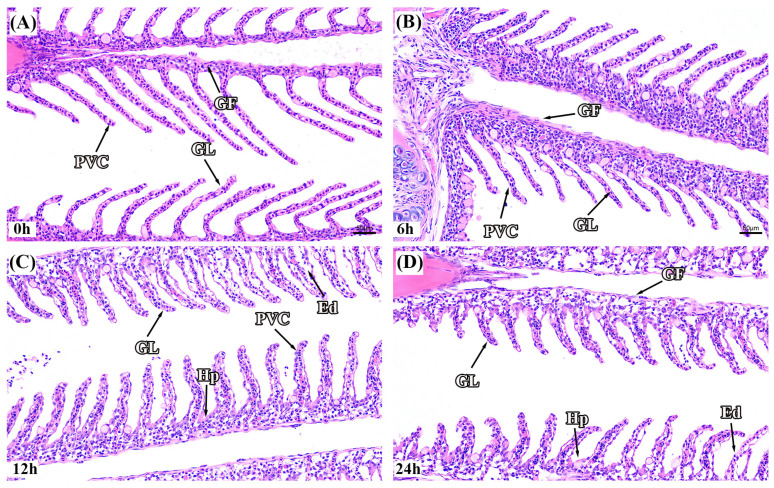
Gill tissue morphological lesions of heat-challenged European perch (*Perca fluviatilis*) visualized via hematoxylin-eosin staining. (**A**) Micrographs from the 0 h control cohort display intact gill filaments (GF), regular secondary lamellae (GL) and intact pavement epithelial cells (PVC). (**B**–**D**) Time-dependent aggravation of branchial lesions after 6 h, 12 h and 24 h thermal exposure, mainly manifested as basal lamellar hyperplasia (Hp) and lamellar swelling edema (Ed). Scale bar = 50 μm.

HE staining of gill tissue revealed that in the control group of *P. fluviatilis*, the gill lamellae were arranged in a regular and orderly pattern, with intact and well-defined structure, and epithelial cells were densely and uniformly organized (Figure 3A). After 6 h of high-temperature stress, slight curling of the lamellae was observed, with no other obvious alterations (Figure 3B). Following 12 h of exposure, partial detachment and vacuolation of the lamellar epithelial cells were evident, along with hyperplasia at the junction between the lamellar base and the primary filament (Figure 3C). After continuous high-temperature exposure for 24 h, the lamellae became generally shorter and thicker, the epithelium separated from the basement membrane, and edema and hyperplasia were observed at the lamellar base, accompanied by irregular bending and disorientation of the lamellae (Figure 3D).

### 3.3. Dynamics of Hepatic Oxidative Stress and Metabolic Enzyme Activities

**Figure 4:**
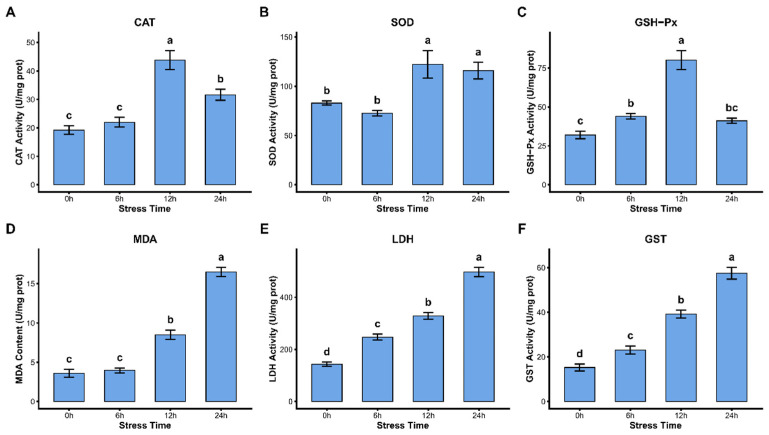
Alterations in key hepatic biochemical biomarkers of European perch (*Perca fluviatilis*) under acute thermal stress. Subgraphs display the levels of six indicators: (**A**) CAT activity, (**B**) SOD activity, (**C**) GSH-Px activity, (**D**) MDA concentration, (**E**) LDH activity, and (**F**) GST activity. Tissue samples were harvested at 0, 6, 12 and 24 h following heat exposure initiation. All values are expressed as mean ± standard error of the mean (SEM), with three biological replicates per time point (*n* = 3). Statistically distinct lowercase labels on each bar denote remarkable discrepancies across all sampling time points (one-way ANOVA coupled with Tukey’s multiple range test, *p* < 0.05). Detailed *F*-statistic values and exact *p*-values for the overall time effects are provided in the main text.

During acute thermal stress, the activities of three key hepatic antioxidant enzymes, including SOD, CAT, and GSH-Px all showed a time-dependent pattern of initial increase followed by a decrease. The temporal variations for all three enzymes were statistically significant (SOD: *F*_(3, 8)_ = 8.49, *p* = 0.0072; CAT: *F*_(3, 8)_ = 24.61, *p* = 0.0002; GSH-Px: *F*_(3, 8)_ = 36.56, *p* < 0.001). After the onset of stress, enzyme activities rose gradually and peaked at 12 h, with levels significantly higher than those in the control group (0 h) (*p* < 0.05). This upregulation suggests that the organism activates antioxidant compensatory defenses to counteract the excess production of reactive oxygen species (ROS) induced by high temperature. When stress duration was extended to 24 h, the activities of all three enzymes decreased significantly compared with their 12 h peaks (*p* < 0.01), indicating that the antioxidant compensatory capacity gradually declines under sustained high temperature stress (Figure 4A–C).

Malondialdehyde (MDA), a core biomarker of lipid peroxidation, showed a continuous, time-dependent increase throughout the stress period (*F*_(3, 8)_ = 136.01, *p* < 0.001). From 0 to 12 h, MDA content increased slowly, corresponding to the compensatory phase of rising antioxidant enzyme activity. This pattern suggests that oxidative damage can be partially buffered by the antioxidant system during this stage. From 12 to 24 h, concomitant with the decrease in antioxidant enzyme activity, the accumulation rate of MDA accelerated markedly, reaching the maximum level at 24 h, which was significantly higher than the baseline control value (*p* < 0.05). This result indicates that oxidative damage continues to intensify during this phase, with progressive lipid peroxidation of hepatic cell membranes (Figure 4D).

For metabolic enzymes, the activities of LDH and GST showed a continuous upward trend over the 24 h stress period, with significant overall alterations across time points (LDH: *F*_(3, 8)_ = 132.02, *p* < 0.001; GST: *F*_(3, 8)_ = 88.66, *p* < 0.001). The activities of both enzymes increased significantly at 6 h and 12 h, and peaked at 24 h, with levels significantly higher than those in the 0 h control group (*p* < 0.05). These changes suggest that hepatic metabolic homeostasis is altered under high temperature stress, with sustained activation of detoxification and anaerobic metabolic pathways (Figure 4E,F).

Correlation analysis with concurrent hepatic histopathological observations showed that the intensification of MDA-mediated lipid peroxidation was highly consistent with the progression of hepatic structural damage. The peak MDA content at 24 h corresponded to a significant increase in the hepatic pathological index at the same time point. Histopathological features such as nuclear karyorrhexis, focal vacuolation, and blurred cell boundaries observed in liver sections are consistent with the physiological effects of membrane structural damage caused by lipid peroxidation. Taken together, these results suggest that oxidative stress-mediated lipid peroxidation may be one of the important mechanisms underlying high temperature-induced hepatic histopathological damage.

### 3.4. Global Transcriptomic Profiling and Systemic ER Stress Signatures

Transcriptome sequencing of six liver and six kidney samples (representing 3 biological replicates × 2 conditions: 0 h control and 24 h heat stress) yielded a total of 81.23 Gb of raw data. After stringent quality control, 80.65 Gb of clean data were retained, corresponding to over 494 million raw reads processed into 38.33–52.05 million clean reads per sample. All sequencing libraries yielded reliable high-quality raw data: valid read proportions surpassed 99.56%, the proportion of bases with Q20 and Q30 quality values maintained levels higher than 98% and 94%, while GC contents fluctuated within the range of 47% to 51%. Further alignment analysis revealed that over 89% of purified clean reads derived from every library could be accurately matched against the reference genome (Supplementary Material S1 Table S2).

**Figure 5:**
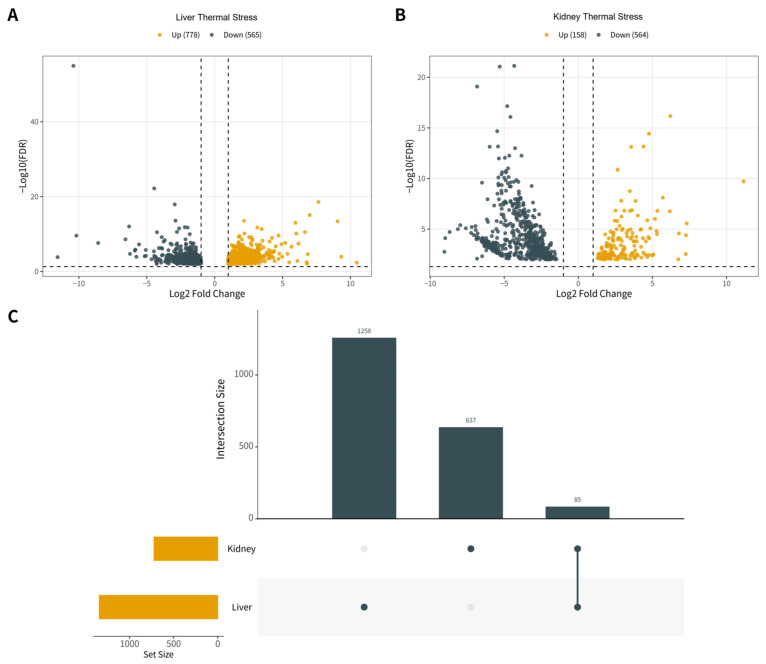
Cross-tissue transcriptomic comparison of hepatic and renal tissues from heat-challenged European perch (*Perca fluviatilis*). (**A**,**B**) Volcano graphs visualize the distribution pattern of genes with significant expression shifts in liver (**A**) and kidney (**B**). The horizontal coordinate corresponds to log_2_-transformed fold change values, whereas the vertical coordinate stands for −log_10_-transformed FDR values. Transcripts satisfying the screening criteria (FDR < 0.05, |Log2 FC| > 1) are colored orange (significantly induced) and dark teal (significantly suppressed), whereas genes without obvious expression variation are rendered gray. (**C**) An UpSet diagram characterizes overlapping and tissue-specific DEG pools across the two organs. The horizontal bar graph on the left quantifies the total DEG count detected in each tissue. The vertical bar graph at the top enumerates transcript sets exclusive to single tissues or co-expressed in both, which are matched to combinations marked by solid dots and linking lines in the matrix panel at the bottom. In panel (**C**), orange bars represent the total DEG set size for each tissue, while dark teal bars denote the intersection size of the respective gene sets.

A total of 1343 DEGs were identified in the liver, with 778 genes upregulated and 565 genes downregulated (Figure 5A). GO analysis revealed that these DEGs were primarily associated with ‘metabolic process’, ‘cellular process’, ‘cellular anatomical entity’, ‘binding’, and ‘catalytic activity’ (Supplementary Material S1 Figure S1). KEGG pathway analysis showed significant enrichment in ‘Metabolic pathways’, ‘Protein processing in endoplasmic reticulum’, ‘Carbon metabolism’, and ‘Oxidative phosphorylation’ (Supplementary Material S1 Figure S2). A total of 722 differentially expressed transcripts were detected within kidney tissues, consisting of 158 induced genes and 564 repressed genes (Figure 5B). GO analysis indicated enrichment in similar terms as the liver, such as ‘cellular process’ and ‘metabolic process’ (Supplementary Material S1 Figure S3). KEGG analysis highlighted pathways like ‘Protein processing in endoplasmic reticulum’, ‘Necroptosis’, ‘Ferroptosis’, and the ‘IL-17 signaling pathway’ (Supplementary Material S1 Figure S4).

To further elucidate the tissue-specific and systemic transcriptomic responses to acute thermal stress in *P. fluviatilis*, an UpSet plot was generated to compare the sets of DEGs between two organs (Figure 5C). The analysis revealed a profound tissue-specific divergence in the magnitude of the stress response. The liver exhibited a substantially broader transcriptomic reprogramming, yielding an overwhelmingly larger number of unique DEGs compared to the kidney. Despite this pronounced tissue specificity, a core intersection of 85 DEGs was commonly regulated in both organs. These 85 core conserved genes orchestrated the systemic damage response (e.g., endoplasmic reticulum stress), whereas the thousands of tissue-specific DEGs implied that the fish adopted highly divergent survival strategies across different vital organs when approaching their physiological limits. Full annotation data of every transcript with differential expression are provided within Supplementary Material S2.

Rather than relying solely on automated pathway enrichment, which may lack sensitivity for small gene sets, we performed an in-depth manual functional annotation of the 85 shared DEGs. Pathway enrichment profiling identified a universally preserved systemic adaptive program that is largely governed by ER stress initiation and subsequent activation of the unfolded protein response (UPR). Specifically, key molecular chaperones and ER quality control sensors, including *hspa5* (GRP78/BiP), *canx* (calnexin), *calr* (calreticulin), and multiple protein disulfide isomerases (*pdia4*, *pdia6*), were consistently upregulated in both tissues. Furthermore, the robust shared induction of chac1, a pro-apoptotic mediator linked to prolonged ER stress, underscores a systemic pathological transition under the thermal and hypoxic crisis (Table 1). These shared signatures highlight that extreme protein denaturation and the subsequent ER stress response constitute the fundamental, organ-independent etiology of thermal injury in *P. fluviatilis*.

### 3.5. Divergent Transcriptomic Reprogramming and Energy Triage in the Liver and Kidney

To further delineate the tissue-specific responses to acute thermal stress, highly significant differentially expressed genes (DEGs) with substantial fold changes were identified in the liver and kidney, covering key functional categories (Table 2).

In the liver, the most prominent upregulated gene was arf2-like (Log2 FC = 10.46), followed by the lipid regulator g0s2 and classic molecular chaperones (e.g., hsp90aa1.2 and hspa1a). A stark metabolic shift was observed, characterized by the upregulation of the gluconeogenic gene pck1 (Log2 FC = 6.77) and the extreme downregulation of the glycolytic gatekeeper gck (Log2 FC = -11.57). Additionally, energy and immune-related genes, such as ucp2 and c1ql2 (complement C1q-like protein 2), were robustly suppressed in the hepatic tissue.

Conversely, the kidney exhibited a distinct transcriptomic reprogramming characterized by intense inflammatory and structural remodeling signals. Pro-inflammatory mediators, including tlr13 (toll-like receptor 13) and il-1b-like (interleukin-1 beta-like), were significantly upregulated, accompanied by extracellular matrix (ECM) regulators such as mmp13-like (collagenase 3-like) and tnxba. Notably, the kidney demonstrated a profound transcriptional shutdown of its fundamental physiological functions. Key osmoregulatory solute carriers (slc38a3b, slc26a6l), nitrogen metabolism enzyme (uox), and major innate immune components (chia-like, c3-like) were among the most severely downregulated genes, indicating a drastic energy triage strategy under thermal crisis.

### 3.6. Quantitative Real-Time PCR Verification of Transcriptome Sequencing Results

**Figure 6:**
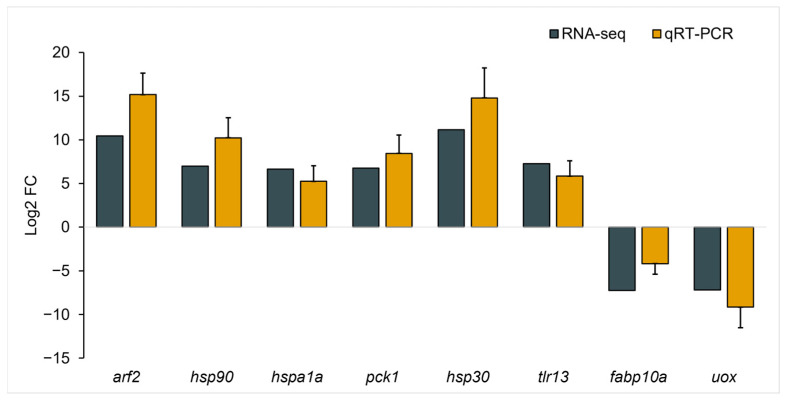
Cross-verification of transcriptome sequencing results of European perch (*Perca fluviatilis*) using quantitative real-time PCR. Expression magnitudes of candidate differentially expressed transcripts were contrasted via two detection approaches: RNA-Seq-derived Log2 FC values and relative expression quantified by qRT-PCR. Genes 1–4 were screened from hepatic samples, while transcripts 5–8 originated from renal tissue. All qRT-PCR quantitative results are displayed as mean ± standard error, with three biological replicates for each group (*n* = 3).

To cross-verify the transcriptional profiling outcomes derived from RNA-Seq, eight transcripts displaying significant expression shifts were chosen for qRT-PCR quantification, namely *arf2-like*, *hsp90aa1.2*, *hspa1a*, *pck1*, *hsp30-like*, *tlr13*, *fabp10a* and *uox*. Parallel comparison of expression patterns acquired via qRT-PCR and high-throughput transcriptome sequencing demonstrated fully matching regulatory tendencies for all selected target genes (Figure 6).

## 4. Discussion

### 4.1. Gill Structural Deformation, Systemic Hypoxia, and Hepatic Metabolic Reprogramming

Gill anatomical integrity is essential for efficient gas exchange and osmoregulation in teleosts [43]. In the present study, histological analysis revealed that acute thermal stress induced progressive pathological lesions in the gills of *P. fluviatilis*, culminating in severe lamellar edema, epithelial detachment, and hyperplasia at 24 h. These morphological deformations substantially thicken the blood-water diffusion barrier, which could limit oxygen uptake and potentially lead to systemic hypoxia [44]. This hypoxia-thermal combined crisis forced a dramatic metabolic reprogramming, particularly in the liver, which acts as the central metabolic hub. Biochemically, this was evidenced by the drastic and continuous elevation of lactate dehydrogenase (LDH) activity, indicating a forced shift from aerobic to anaerobic glycolysis to sustain cellular energy production. Transcriptomic data perfectly corroborated this physiological shift: the profound downregulation of *gck* alongside the robust upregulation of *pck1* suggests that the liver effectively arrested its own glucose utilization. Instead, it transformed into a gluconeogenic organ, actively recycling the accumulating systemic lactate generated by hyperactive LDH. Furthermore, the significant induction of the hypoxia-responsive mitochondrial protector *higd1a* at the transcriptional level strongly suggest that gill-induced hypoxia was the primary driver of this hepatic metabolic restructuring. Notably, *higd1a* plays a critical role in maintaining mitochondrial homeostasis under hypoxic conditions [45]. This molecular evidence perfectly aligns with the widely recognized oxygen- and capacity-limited thermal tolerance (OCLTT) hypothesis, which posits that the initial vulnerability of fish to extreme heat is largely driven by a mismatch between cellular oxygen demand and the limited capacity of the cardiorespiratory system to deliver oxygen [18]. Importantly, because the significantly elevated LDH activity was quantified strictly from intact hepatic tissue homogenates rather than from systemic circulating plasma, its continuous rise robustly reflects active intracellular metabolic re-routing toward anaerobic glycolysis. This interpretation is further supported by corresponding histological observations, which showed localized cellular damage but lacked evidence of massive, organ-wide hepatolysis that would passively leak massive intracellular enzymes.

### 4.2. Energetic Trade-Offs, Oxidative Stress, and Structural Collapse in the Liver

Under extreme thermal stress, the massive synthesis of heat shock proteins (e.g., HSP70, HSP90) is critical for refolding denatured proteins but imposes a tremendous ATP burden on the hepatic tissue [46]. To maximize ATP yield, the liver may adopt a potentially detrimental energetic trade-off via substantial downregulation of ucp2. Suppression of this mitochondrial “leak-valve” could boost oxidative phosphorylation efficiency, which may in turn lead to excessive production of reactive oxygen species (ROS) overproduction [47]. Our biochemical assays captured changes consistent with this ROS surge: the primary antioxidant defenses (SOD and CAT) showed a robust initial response, peaking at 12 h, but declined significantly at 24 h, indicating gradual exhaustion of the antioxidant system. With the attenuation of antioxidant defense capacity, ROS may continuously attack cellular macromolecules and aggravate lipid peroxidation, which aligns with the sustained accumulation of MDA observed in our assays [48]. Hepatic H&E staining revealed typical pathological changes at 24 h, including marked nuclear pyknosis, focal vacuolation, and disrupted tissue architecture. Meanwhile, the transcriptomic level of *aspp2-like*, a potent p53-mediated pro-apoptotic factor, was upregulated in bulk liver tissue [49]. This temporal change in gene expression coincided with the increased occurrence of nuclear fragmentation observed in histology, which only reflects a general stress trend at the tissue level, rather than direct evidence of cellular co-localization. While the antioxidant collapse post-12 h was severe, the acceleration rate of MDA accumulation did not steepen as sharply as anticipated. This non-linear dynamic fundamentally suggests that the primary substrate for peroxidation—intact polyunsaturated lipids within the cellular membranes—was being progressively depleted, a phenomenon intrinsically coupled with severely damaged hepatocytes entering the terminal phase of irreversible necrosis.

### 4.3. Systemic ER Stress and Divergent Organ-Specific Apoptotic Strategies

Based on conventional pathway enrichment results, we manually annotated the shared core DEGs (Table 1) to reconstruct potential regulatory cascades of thermal injury. Our transcriptomic data suggest that systemic endoplasmic reticulum (ER) stress and activation of the unfolded protein response (UPR) represent a core molecular response underlying acute thermal injury in *P. fluviatilis*. The intracellular accumulation of heat-denatured proteins induced by elevated temperature universally activated the unfolded protein response (UPR) across both vital organs. This response was highlighted by the highly consistent upregulation of the UPR master regulator *hspa5* (BiP), alongside a suite of ER quality control elements including *calr*, *pdia4*, and *canx* [22].

Furthermore, the shared upregulation of *pfkfb3* (a potent glycolytic activator) may suggest a systemic metabolic push to generate rapid ATP via glycolysis to support the energy demand of enhanced protein-refolding machinery [50].

Most notably, while the core ER stress response was conserved between the two organs, the downstream cell-fate regulatory patterns diverged significantly, as exemplified by the expression pattern of *chac1*. CHAC1 is widely recognized as a pro-apoptotic mediator triggered by prolonged, unresolved ER stress [51]. In the liver, *chac1* was upregulated, indicating that severe heat damage may drive hepatocytes towards programmed cell death. This molecular change aligns well with our H & E staining observations of nuclear fragmentation in liver tissue.

Conversely, *chac1* was significantly downregulated in the kidney. This downregulation, coupled with the suppressed expression of mitochondrial *diablo* [52], is consistent with a potential strict anti-apoptotic strategy deployed by renal tissue. By actively attenuating death signals even under severe ER stress, the kidney may prioritize maintaining its basic structural and functional integrity under thermal stress.

### 4.4. Compensatory Rescue Mechanisms: Membrane Remodeling and Lipid Sequestration

In a critical compensatory response to counteract the catastrophic structural damage and lipid peroxidation, the surviving hepatic cells activated distinct compensatory pathways. The most strikingly upregulated transcript in the liver was *arf2-like* (over 1000-fold increase), a crucial regulator of intracellular vesicular trafficking [53]. Its baseline FPKM expression in control groups was extremely low, magnifying the observed fold change. The massive induction of *arf2-like* reflects an urgent, high-priority cellular response to accelerate targeted vesicle transport for membrane patching and structural repair. Furthermore, the synergistic upregulation of *g0s2* and *gstk1* uncovered a highly coordinated detoxification strategy. The *g0s2* gene is highly conserved in vertebrates and functions as a master regulator of lipid storage and mobilization [54]. By promoting lipid droplet formation, G0S2 likely sequesters toxic, oxidized lipid intermediates (MDA precursors) away from sensitive organelles. Subsequently, these compartmentalized toxins can be neutralized by the intensely activated phase II detoxification system, supported by both the robust elevation of GST enzyme activity and the drastic transcriptional induction of mitochondrial *gstk1* [55].

### 4.5. Renal Energy Triage and Systemic Inflammatory Storm

While the liver actively engaged in metabolic reprogramming and membrane repair, the transcriptomic profile of the kidney—the primary immune, hematopoietic, and osmoregulatory organ in teleosts [56]—revealed a profound strategy of “energy triage.” To conserve limited ATP for essential survival pathways, the kidney nearly completely suspended its normal physiological duties. This was evidenced by the steep downregulation of major solute carriers (e.g., *slc38a3b*, *slc26a6l*) and uox, alongside the shutdown of highly energy-consuming innate immune defenses (e.g., *c3-like*, *nattectin*). Concurrently, the kidney became the epicenter of a heat-induced inflammatory storm. The release of damage-associated molecular patterns (DAMPs) from necrotic cells likely triggered the intense upregulation of *tlr13* and the pro-inflammatory master regulator *il-1b-like* [57]. Histological distress was molecularly mirrored by the rapid degradation of the extracellular matrix (ECM), indicated by elevated *mmp13-like* and *tnxba* [58]. However, to avoid severe tissue necrosis and renal structural breakdown, renal tissue may suppress the mitochondrial pro-apoptotic factor *diablo* to mitigate excessive cell death. Meanwhile, the significant upregulation of thyroxine 5-deiodinase-like potentially acts to lower local basal metabolic rate by modulating thyroid hormone signaling [59]. This suggests a state of functional hibernation, where the kidney sacrificed systemic homeostasis to weather the lethal thermal crisis. While this ‘energy triage’ strategy confers a short-term survival advantage by preventing immediate ATP depletion and total organ liquefaction [60,61], its long-term consequences are inherently detrimental. The prolonged suppression of osmoregulatory and innate immune pathways leaves the fish highly vulnerable to severe systemic osmotic imbalance and secondary opportunistic infections [62,63]. Furthermore, the localized immune suppression combined with massive DAMPs release triggers a lethal intra-renal inflammatory storm [64,65]. This functional trade-off may explain the macroscopic phenotypic paradox observed in our study: why the cumulative mortality of the fish continues to climb progressively over time, even though pro-apoptotic pathways are strongly suppressed in renal cells at the molecular level. Overall, such local organ protection may serve as an acute compensatory response that favors temporary cell survival, which could trigger adverse systemic impacts upon prolonged heat stress.

### 4.6. Comparative Perspectives on Teleost Thermal Survival Paradigms

When evaluating these pronounced organ-specific transcriptional responses, it is critical to contextualize the thermal survival phenotype of *P. fluviatilis* within a broader comparative physiological framework. Recent transcriptomic studies of other economically important teleost species have identified both conserved transcriptional signatures and highly divergent response patterns across heat stress pathways.

For instance, in the cold-water salmonid rainbow trout, moderate heat stress is associated with prominent transcriptional upregulation of genes involved in protein metabolism and endoplasmic reticulum (ER) protein processing pathways. However, the hepatic transcriptional response in trout is dominated by signatures of ER-associated degradation and upregulation of calpain, a putative upstream regulator of apoptosis [24]. In contrast, hepatic transcriptional profiles in perch show concurrent upregulation of genes linked to *chac1*-mediated apoptosis and a pronounced shift toward anaerobic glycolysis, alongside putative signatures of vesicular membrane regulation mediated by *arf2-like*.

Furthermore, comparison of *P. fluviatilis* with warm-tolerant cyprinid species such as grass carp reveals distinct patterns of metabolic remodeling. Elevated temperatures induce widespread transcriptional alterations in grass carp liver and brain, including upregulation of lipid metabolism-related genes, downregulation of fatty acid synthesis pathways, and perturbed expression of immune gene sets [26]. While *P. fluviatilis* exhibits comparable lipid remodeling signatures (supported by upregulation of g0s2, a gene implicated in lipid droplet sequestration) and transcriptional downregulation of innate immune pathways in renal tissue, these changes correspond to a distinct transcriptional profile consistent with an “energy triage” phenotype in the kidney. Transcriptional attenuation of energy-intensive solute carriers and downregulation of pro-apoptotic signaling pathways are associated with preserved baseline structural integrity of renal tissue under thermal stress.

Finally, in contrast to the benthic marine Chinese tongue sole, which exhibits rapid induction of central neuroendocrine heat responses via transcriptional activation of cortisol synthesis, neuroactive ligand–receptor interactions, and TGF-beta/JAK-STAT signaling pathways in the brain [25], the thermal survival limits of *P. fluviatilis* appear more closely associated with tissue-specific functional perturbations in peripheral metabolic and osmoregulatory organs.

Collectively, this cross-species comparison contextualizes the evolutionary specificity of the perch’s extreme thermal stress response profile, which aligns with its relatively constrained thermal buffering capacity as inferred from transcriptional stress signatures.

### 4.7. Study Limitations and Future Perspectives

While this integrated study provides a profound, multi-level evaluation of the physiological thresholds and transcriptomic reprogramming boundaries of *P. fluviatilis* under extreme heat, several critical limitations must be transparently acknowledged to guide future research. First, our highly controlled laboratory acute heating model (a linear 2 °C/h ramp to 31 °C) successfully and deliberately isolates the pure physiological variable of critical thermal tolerance. However, this simplified model inherently fails to fully replicate the multifactorial, complex dynamics of real-world aquaculture heatwaves, which typically involve slower, deeply fluctuating thermal cycles severely compounded by interactive stressors such as environmental hypoxia, intense algal blooms, and elevated ammonia toxicity.

Second, our transcriptomic architecture focused exclusively on contrasting the 0 h absolute baseline with the 24 h terminal survival inflection point. While this strategic chronological pairing successfully captured the definitive, terminal “cell-fate decision” networks dictating species survival or systemic collapse, the absence of intermediate temporal sequencing nodes (e.g., at 6 h and 12 h) critically limits our ability to smoothly map the continuous, early-wave dynamic trajectory of gene activation and suppression.

Third, thermal tolerance in teleosts exhibits severe ontogenetic variation. The extreme physiological strategies identified herein—such as profound hepatic metabolic re-routing and strict renal energy triage—reflect the mature adaptations of juvenile/adult specimens. These mechanisms cannot be directly extrapolated to highly vulnerable embryonic, larval, or actively spawning adult stages, which often possess dramatically narrower thermal tolerance windows and entirely different metabolic priorities.

Finally, while our dual-organ RNA-Seq approach successfully identified thousands of differentially expressed genes, these data remain fundamentally correlative. The profound up-regulation of specific rescue candidates—such as the massive induction of the vesicular trafficking regulator *arf2-like* for putative membrane repair, and the lipid-droplet regulator *g0s2* for theoretical toxic lipid sequestration—demands rigorous downstream verification. Future investigations must pivot towards in vivo functional validation, utilizing advanced targeted gene-knockdown techniques such as RNA interference (RNAi) or CRISPR/Cas9, integrated with deep proteomics, to definitively confirm the physiological protective roles of these biomarkers in breeding more thermally resilient perch strains.

## 5. Conclusions

In summary, this study provides a comprehensive, multi-level evaluation of the acute heat stress response in *Perca fluviatilis*, integrating histopathological, biochemical, and dual-organ transcriptomic approaches. Our findings suggest that acute thermal exposure induces progressive structural lesions in the gills and liver, accompanied by a hepatic oxidative crisis characterized by lipid peroxidation and eventual antioxidant exhaustion. At the molecular level, both the liver and kidney share a systemic endoplasmic reticulum stress and unfolded protein response signature, yet they appear to execute highly divergent physiological strategies. The liver shows evidence of a profound metabolic shift toward anaerobic glycolysis and gluconeogenesis, prioritizing cell-membrane repair via *arf2-like* vesicular trafficking and *g0s2*-mediated lipid droplet sequestration. Conversely, the kidney shows evidence of an energy-conservation strategy, suppressing high-energy-consuming osmoregulatory and immune functions while actively silencing pro-apoptotic pathways to sustain basal cellular survival. Collectively, these integrated insights may improve our understanding of organ-specific adaptations and systemic survival limits in European perch under acute thermal stress. From an applied perspective, the consistently upregulated ER stress chaperones (*hspa5*, *calr*, *canx*) and the compensatory repair gene *arf2-like* represent promising molecular markers for evaluating thermal tolerance in selective breeding programs for *P. fluviatilis*. For future breeding efforts targeting strains capable of surviving prolonged hot summers in southern aquaculture habitats, complementary joint assessments integrating genome-wide association studies (GWAS) or chronic thermal tolerance-linked SNP markers are still indispensable. Crucially, the key responsive genes and metabolic networks identified herein offer promising candidate molecular markers to facilitate selective breeding programs for heat-resistant perch strains, thereby supporting the strategic expansion of its aquaculture across wider temperature ranges and diverse regions in China.

## Figures and Tables

**Table 1:** Key shared differentially expressed genes (DEGs) highlighting systemic ER stress and divergent apoptotic strategies in the liver and kidney of European perch (*Perca fluviatilis*) under acute thermal stress.

Functional Category	Gene Symbol	Gene Description	Log2 FC (Liver)	Log2 FC (Kidney)
ER Stress & Protein Folding	*hspa5*	Endoplasmic reticulum chaperone BiP	4.05	3.86
	*calr*	Calreticulin	4.17	3.01
	*pdia4*	Protein disulfide-isomerase A4	2.66	2.81
	*canx*	Calnexin	1.84	2.25
Metabolic Re-routing	*pfkfb3*	6-phosphofructo-2-kinase/fructose-2,6-biphosphatase 3	2.37	2.69
Apoptotic Signaling	*chac1*	Glutathione-specific gamma-glutamylcyclotransferase 1	1.10	−2.67

**Table 2:** Key differentially expressed genes highlighting tissue-specific functional reprogramming in the liver and kidney of European perch (*Perca fluviatilis*) under thermal stress.

Tissue	Functional Category	Gene Name/Symbol	Regulation	Log2 FC	FDR
Liver	Membrane & Lipid Remodeling	*arf2-like*	Up	10.46	4.37 × 10^−3^
		*g0s2*	Up	9.05	3.97 × 10^−14^
	Stress Response & Chaperones	*hsp90aa1.2*	Up	7.00	8.12 × 10^−16^
		*hspa1a*	Up	6.65	3.12 × 10^−11^
		*higd1a*	Up	5.37	1.95 × 10^−5^
		*ucp2*	Down	−6.57	2.59 × 10^−09^
	Metabolic Reprogramming	*pck1*	Up	6.77	3.36 × 10^−3^
		*gck*	Down	−11.57	1.48 × 10^−4^
	Immunity & Apoptosis	*map2k6*	Down	−4.34	1.26 × 10^−4^
		*c1ql2*	Down	−10.41	1.17 × 10^−55^
Kidney	Stress Response & Chaperones	*hsp30-like*	Up	11.15	1.91 × 10^−10^
		*serpinh1b*	Up	5.31	3.13 × 10^−5^
		*ucp1*	Down	−6.83	8.31 × 10^−3^
		*diablo*	Down	−6.88	9.77 × 10^−5^
	Inflammation & Immunity	*tlr13*	Up	7.26	2.84 × 10^−3^
		*il-1b-like*	Up	5.34	1.54 × 10^−7^
		*c3-like*	Down	−6.54	1.49 × 10^−4^
		*nattectin*	Down	−7.97	4.04 × 10^−6^
		*chia-like*	Down	−8.98	7.73 × 10^−5^
	Tissue & ECM Remodeling	*tnxba*	Up	6.21	6.83 × 10^−17^
		*mmp13-like*	Up	6.18	1.72 × 10^−7^
	Metabolic Shutdown	*dio5-like*	Up	5.17	9.77 × 10^−7^
		*dgat2*	Down	−6.35	2.18 × 10^−4^
		*fbp1b*	Down	−6.92	1.23 × 10^−5^
		*fabp10a*	Down	−7.24	2.38 × 10^−5^
	Transport & Excretion	*uox*	Down	−7.19	6.20 × 10^−6^
		*slc26a6l*	Down	−6.50	2.58 × 10^−10^
		*slc38a3b*	Down	−9.03	1.72 × 10^−3^

## Data Availability

All raw transcriptome sequencing data generated in this thermal stress experiment have been submitted to the NCBI Sequence Read Archive (SRA) public database, which can be accessed via the accession number PRJNA1470504 and PRJNA1346549. Relevant datasets supporting the findings of this study are available from the corresponding author upon reasonable request.

## Supplementary Materials

The following supporting information can be downloaded at: https://www.mdpi.com/article/10.3390/ani16132007/s1, Table S1: Primers used in the study. Table S2: Summary of RNA-Seq data quality control. Figure S1: GO enrichment analysis of differentially expressed genes (DEGs) in liver. Figure S2: KEGG enrichment analysis of differentially expressed genes (DEGs) in liver. Figure S3: GO enrichment analysis of differentially expressed genes (DEGs) in kidney. Figure S4: KEGG enrichment analysis of differentially expressed genes (DEGs) in kidney. Supplementary Material S2: Gene expression data of differentially expressed genes under acute heat stress.

## Abbreviations

The abbreviations listed below are adopted throughout the manuscript for concise academic description:

ANOVA	One-way analysis of variance
ARF2-like	ADP-ribosylation factor 2-like
ASPP2-like	Apoptosis-stimulating of p53 protein 2-like
ATF4	Activating transcription factor 4
BiP (HSPA5)	Binding immunoglobulin protein/Endoplasmic reticulum chaperone BiP
C1QL2	Complement C1q-like protein 2
C3-like	Complement C3-like
CALR	Calreticulin
CANX	Calnexin
CAT	Catalase
CHAC1	Glutathione-specific gamma-glutamylcyclotransferase 1
CHIA-like	Chitinase acidic-like
DAMPs	Damage-associated molecular patterns
DEGs	Differentially expressed genes
DGAT2	Diacylglycerol O-Acyltransferase 2
DIABLO	Direct IAP binding protein with low pI
DIO5-like	Deiodinase 5-like/Type 5 deiodinase-like
ECM	Extracellular matrix
ER	Endoplasmic reticulum
FABP10a	Fatty acid-binding protein 10a
FBP1b	Fructose-bisphosphatase 1b
FDR	False discovery rate
FPKM	Fragments Per Kilobase of transcript per Million mapped reads
G0S2	G0/G1 switch gene 2
GCK	Glucokinase
GO	Gene Ontology
GSH-Px	Glutathione peroxidase
GST	Glutathione S-transferase
GSTK1	Glutathione S-transferase kappa 1
H & E	Hematoxylin and eosin
HIF-1	Hypoxia-inducible factor 1
HIGD1A	HIG1 hypoxia inducible domain family member 1A
HSP	Heat shock protein
HSPA1a	Heat shock 70 kDa protein 1A
HSP90aa1.2	Heat shock protein 90 alpha family class A member 1.2
IL-1B-like	Interleukin-1 beta-like
KEGG	Kyoto Encyclopedia of Genes and Genomes
LDH	Lactate dehydrogenase
MAP2K6	Mitogen-activated protein kinase kinase 6
MDA	Malondialdehyde
MMP13-like	Matrix metalloproteinase 13-like
OCLTT	Oxygen- and capacity-limited thermal tolerance
PCK1	Phosphoenolpyruvate carboxykinase 1
PDIA4/6	Protein disulfide-isomerase A4/6
PFKFB3	6-phosphofructo-2-kinase/fructose-2,6-biphosphatase 3
qRT-PCR	Quantitative real-time PCR
ROS	Reactive Oxygen Species
SERPINH1b	Serpin family H member 1b
SLCs	Solute carrier family members (e.g., slc38a3b, slc26a6l)
SOD	Superoxide dismutase
TLR13	Toll-like receptor 13
TNXBA	Tenascin XB
UCP1/2	Uncoupling protein 1/2
UPR	Unfolded Protein Response
UOX	Urate oxidase
