# Supplementary material for: Integrated Physiological and Transcriptomic Analyses Suggest Key Adaptive Mechanisms of European Perch (Perca fluviatilis) to Acute Heat Stress
Source: Animals (Basel). 2026 Jul 1;16(13):2007. doi: 10.3390/ani16132007 (PMC13359654; doi:10.3390/ani16132007)
Supplement: Supplementary file 1 [file animals-16-02007-s001.zip › Supplementary Material S1.pdf]

Table S1. Primers used in the study

Primer name	Sequence(5′-3′)	Amplification efficiency
β-actin-F	AACCAACGCCCAACAACTTC	96%
β-actin-R	ACGTTCTCCTTCATCGTTCCAG	
hspa1a-F	TAACCTGTGGGTGACTTCGC	101%
hspa1a-R	GCTGGGGATGCTCTTCCTAC	
hsp90-F	CTCTTGCGTCCAGAGCGTTA	98%
hsp90-R	TGAGCAGAACCTCCCCAAAC	
arf2-F	GTGCTACCCTGCATCTGTGT	102%
arf2-R	GATGTGCCAACACTGGAGGA	
pck1-F	ATGAAAGGCCGCACCATGTA	95%
pck1-R	CAGCGGACAAACTCTCCTGT	
hsp30-F	AAGATGCTGTGTTCCCGAGG	95%
hsp30-R	CTCTGCAGTAGATCCCGCTG	
tlr13-F	GTGCTCTTTGTTGCGACTGG	98%
tlr13-R	GTGTTGGATCGCATGGCTTC	
fabp10a-F	CATGGCAGGTCTACTCCCAG	105%
fabp10a-R	CCGACGGTAAAGGAGTTGGT	
uox-F	AACAGGCTACGGCAAGAACA	102%
uox-R	TTCTTGATGGTGTCGGTGGG	

Table S2. Summary of RNA-Seq data quality control

SampleID	rawReads	cleanReads	mapped ratio	Q30	GC
Control_Liver_Rep1	52,227,836	52,051,750	92.30%	96.36%	50.10%
Control_Liver_Rep2	48,841,942	48,685,776	92.74%	94.80%	49.87%
Control_Liver_Rep3	40,681,298	40,548,378	92.70%	96.13%	50.22%
Control_Kidney_Rep1	50,297,400	50,129,398	92.93%	96.35%	50.71%
Control_Kidney_Rep2	45,153,258	45,022,910	93.12%	96.35%	50.19%

Control_Kidney_Rep3	46,045,310	45,894,432	91.04%	96.11%	48.36%
Exp_Liver_Rep1	50,335,196	50,174,558	89.51%	96.29%	50.43%
Exp_Liver_Rep2	40,789,046	40,682,256	92.10%	96.00%	49.21%
Exp_Liver_Rep3	38,434,576	38,332,172	92.93%	96.08%	49.29%
Exp_Kidney_Rep1	49,607,034	49,390,250	90.61%	94.79%	47.64%
Exp_Kidney_Rep2	39,734,310	39,606,568	90.21%	96.02%	47.71%
Exp_Kidney_Rep3	39,597,138	39,470,332	90.42%	95.91%	47.69%

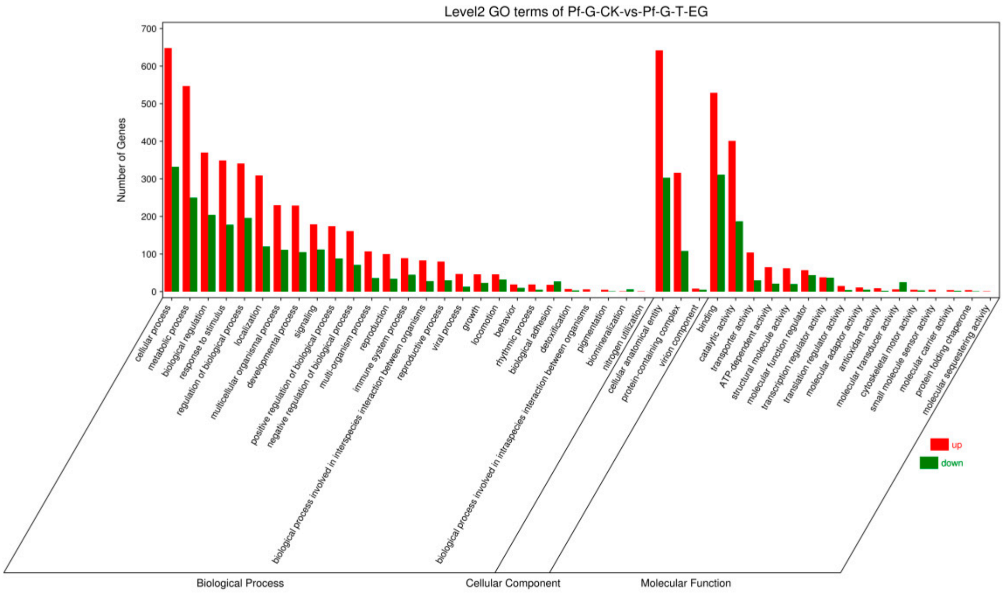

Figure S1. GO enrichment analysis of differentially expressed genes (DEGs) in liver. The most significantly enriched GO terms in biological process (BP), cellular component (CC), and molecular function (MF) categories.

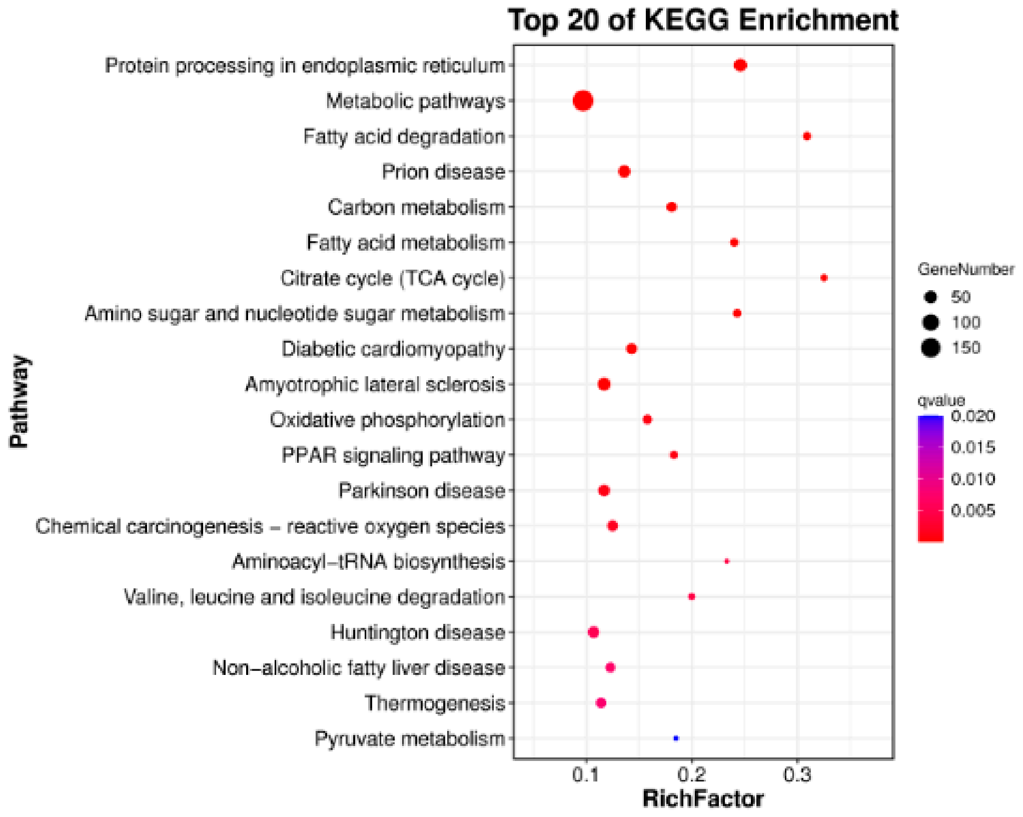

Figure S2. KEGG enrichment analysis of differentially expressed genes (DEGs) in liver. The vertical axis represents the name of the term or pathway, and the horizontal axis represents the rich factor. The size of the dot indicates the number of DEGs enriched in that term, and the color represents the range of the false discovery rate (FDR) corrected P-value (Q-value).

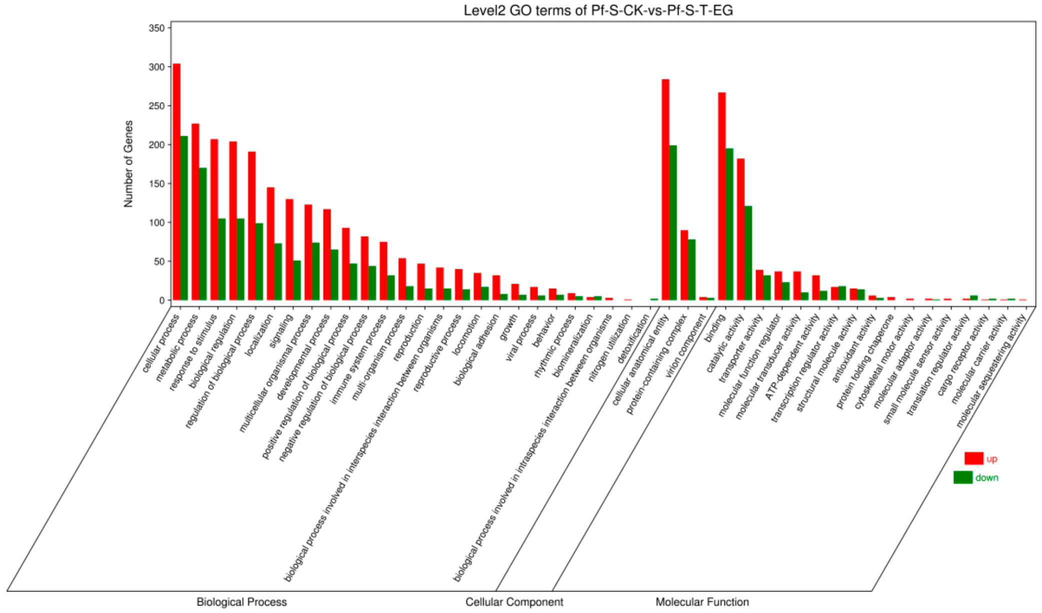

Figure S3. GO enrichment analysis of differentially expressed genes (DEGs) in kidney. The most significantly enriched GO terms in biological process (BP), cellular component (CC), and molecular function (MF) categories.

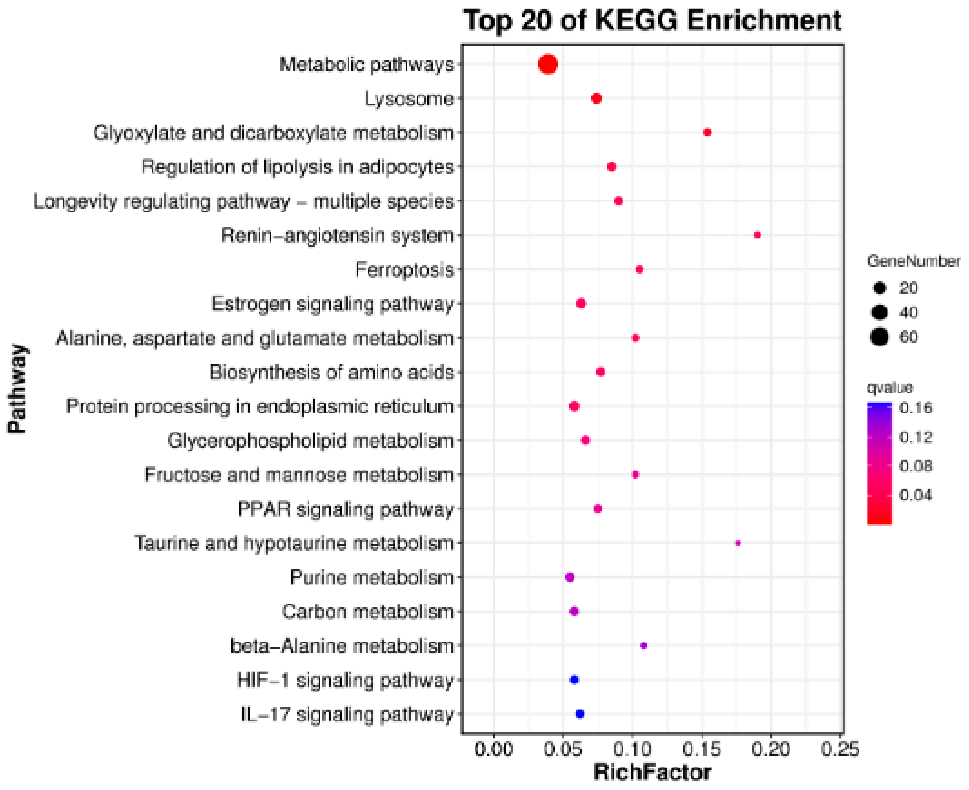

Figure S4. KEGG enrichment analysis of differentially expressed genes (DEGs) in kidney. The vertical axis represents the name of the term or pathway, and the horizontal axis represents the rich factor. The size of the dot indicates the number of DEGs enriched in that term, and the color represents the range of the false discovery rate (FDR) corrected P-value (Q-value).
